# Impact of posttraumatic stress disorder on incident cardiovascular outcomes in women veterans: a retrospective longitudinal study

**DOI:** 10.1016/j.eclinm.2026.104197

**Published:** 2026-09-08

**Authors:** Ramin Ebrahimi, Paul A. Dennis, Carlos A. Alvarez, A. Laurie Shroyer, Jean C. Beckham, Jennifer A. Sumner

**Affiliations:** a Department of Medicine, University of California, Los Angeles, CA, USA; b Veterans Affairs Greater Los Angeles Healthcare System, Los Angeles, CA, USA; c Department of Population Health Sciences, Duke University School of Medicine, Durham, NC, USA; d Durham VA Medical Center, Durham, NC, USA; e Department of Pharmacy Practice, Texas Tech University Health Sciences Center, Dallas, TX, USA; f Department of Research, VA North Texas Health Care System, Dallas, TX, USA; g Department of Surgery, Renaissance School of Medicine, Stony Brook University, Stony Brook, NY, USA; h Northport VA Medical Center, Northport, NY, USA; i Department of Psychiatry and Behavioral Sciences, Duke University School of Medicine, Durham, NC, USA; j Department of Psychology, University of California, Los Angeles, CA, USA

**Keywords:** Women, Veterans, PTSD, Cardiovascular disease

## Abstract

**Background:**

Women veterans are a growing—yet understudied—population with a high psychiatric and cardiovascular disease (CVD) risk burden. This study evaluated the association of posttraumatic stress disorder (PTSD) with a wide range of incident CVD outcomes in a large, nationally representative cohort of women veterans.

**Methods:**

In this retrospective longitudinal study, Veterans Health Administration (VHA) electronic health records were used to identify women veterans aged ≥18 years receiving care from 1/1/2000 to 12/31/2019 in the USA. Women with prior CVD at baseline were excluded. Participants with PTSD (*n* = 101,448) were matched 1:1 to women without PTSD on demographic, clinical, and healthcare utilization factors. Diagnoses were based on International Classification of Diseases codes. Cox proportional hazards models estimated hazard ratios (HRs) and 95% confidence intervals (CIs) for associations of PTSD with an incident CVD composite (IHD, stroke/transient ischemic attack [TIA], heart failure [HF], atrial fibrillation, peripheral arterial disease, pulmonary hypertension [PHTN], venous thromboembolism [VTE], and aortic valve stenosis [AS]), and each component outcome.

**Findings:**

The analytic sample comprised 202,896 women veterans (mean age = 39.1 years). Over approximately 6 years of follow-up, PTSD was associated with a greater rate of developing the CVD composite (HR = 1.24, 95% CI: 1.20–1.28). PTSD was also associated with greater incidence of IHD (HR = 1.30, 95% CI: 1.23–1.37), stroke/TIA (HR = 1.29, 95% CI: 1.21–1.38), HF (HR = 1.10, 95% CI: 1.02–1.18), PHTN (HR = 1.17, 95% CI: 1.02–1.35), VTE (HR = 1.34, 95% CI: 1.23–1.45), and AS (HR = 1.23, 95% CI: 1.03–1.46). Particularly strong associations were observed in women <40 years and in American Indian/Alaska Native and Hispanic/Latina women.

**Interpretation:**

PTSD is associated with a broad spectrum of CVD outcomes in women veterans. Results also indicate associations of PTSD with PHTN and AS, novel outcomes in the PTSD-CVD literature—including in women veterans. Targeted studies are needed to better understand underlying mechanisms.

**Funding:**

This research was funded by a grant from the Department of Defense Office of Congressionally Directed Medical Research Programs.The funder had no role in the study design, data collection, analysis, interpretation, or writing of the manuscript.

**Research in context**

**Evidence before this study**

Posttraumatic stress disorder has been linked to cardiovascular disease, mainly coronary artery disease, for over five decades. However, most of the research on the subject has been limited to men. In this project we searched the PubMed/Medline database from 1970 to January 2026 and used the following terms: posttraumatic stress disorder, stress, PTSD, cardiovascular disease, CVD, coronary artery disease, CAD, ischemic heart disease, myocardial infarction, heart attack, stroke, atrial fibrillation, heart failure, venous thromboembolism, deep venous thrombosis, pulmonary embolism, peripheral arterial disease, pulmonary hypertension, aortic stenosis, women, and females. The search was limited to English language.

**Added value of this study**

In this large longitudinal retrospective analysis of the USA women veterans’ electronic health records we found a significant association of PTSD with a large set of cardiovascular diseases beyond ischemic heart disease (IHD) and stroke in a relatively young population of women veterans with a mean age of 39.1 years. Specifically, we found significant PTSD association with heart failure, venous thromboembolism, atrial fibrillation and peripheral arterial disease, that has been rarely investigated, and aortic stenosis and pulmonary hypertension, that to our knowledge have never been investigated before. In addition, we found particularly strong associations in women <40 years and in American Indian/Alaska Native and Hispanic/Latina women.

**Implications of all the available evidence**

Our findings call for earlier assessment of CVD risk in USA women veterans with PTSD who are at particularly high risk of CVD. Further research is indicated to investigate the potential need for earlier CVD risk assessment in non-veteran women and men with PTSD, to verify the association of PTSD with aortic stenosis and pulmonary hypertension, to investigate the potential mechanisms related to such associations, and to assess the impact of tailored interventions aimed at reducing cardiovascular risk in this high-risk population.

## Introduction

Trauma exposure is common, with global estimates suggesting that approximately 70% of individuals will experience at least one traumatic event in their lifetime.^1^ Posttraumatic stress disorder (PTSD) is an often chronic and disabling mental disorder that can onset after trauma exposure, with far-reaching consequences for health.^2^ PTSD has increasingly emerged as a risk factor associated with cardiovascular disease (CVD), with evidence suggesting it may act as an independent risk factor for some outcomes, even after adjustment for traditional risk factors and comorbidities.^3^ Meta-analytic evidence from longitudinal studies has linked PTSD to heightened incidence of coronary heart disease and stroke, with pooled hazard ratios of approximately 1.6.^4^^,^^5^ However, the literature examining PTSD and onset of other cardiovascular outcomes is comparatively less developed. Relatively few studies have investigated the association of PTSD with cardiovascular outcomes such as venous thromboembolism (VTE),^6^, ^7^, ^8^ atrial fibrillation,^8^^,^^9^ heart failure,^8^^,^^10^^,^^11^ and peripheral vascular disease.^10^ Furthermore, despite growing appreciation of the public health burden of conditions such as aortic valve stenosis (AS) and pulmonary hypertension (PHTN),^12^^,^^13^ these cardiovascular outcomes have yet to be examined with respect to PTSD. Thus, examining the potential association of PTSD with a broader range of cardiovascular outcomes, beyond heart disease and stroke, is important to advance the field.

Moreover, despite growing evidence that PTSD may lead to onset of various cardiovascular conditions, research in women veterans—a high risk population^14^—is limited. Women veterans are the fastest growing group within users of the Veterans Health Administration (VHA), nearly tripling from 159,810 in 2000 to 439,791 in 2015.^15^ Furthermore, the number of women veterans is projected to increase to 3.3–3.4 million by 2040, representing nearly 18% of all living United States veterans.^16^ Notably, women veterans are generally younger and more racially and ethnically diverse than men veterans,^15^ yet they carry a particularly high burden of psychiatric (e.g., PTSD, depression) and traditional (e.g., hypertension, hyperlipidemia, and smoking) CVD risk factors compared to civilian women or men veterans.^14^ Despite this elevated CVD risk burden and greater CVD-related mortality compared with civilian women,^14^^,^^17^ women veterans have been underrepresented in research on PTSD and CVD. As such, targeted CVD risk screening and reduction, specifically aiming to diagnose and treat such risk factors, in women veterans has been identified as a key priority within the VHA.^18^ Although we demonstrated that PTSD is associated with heightened incidence of ischemic heart disease (IHD)^19^ and stroke/transient ischemic attack (TIA)^20^ in large samples of women veterans, understanding the potential impact of PTSD on a wide range of CVD outcomes—including less well-investigated conditions—in women veterans is important, yet remains unknown.

In the current study, we addressed this gap by examining the extent to which PTSD was associated with incidence of a CVD composite, including IHD, stroke/TIA, HF, AF, peripheral arterial disease (PAD), PHTN, VTE, and AS, in women veterans. In addition, we investigated such associations with each CVD component, and we performed stratified analyses based on age and race/ethnicity to evaluate potential differences in the PTSD-CVD composite relation, given the predominantly younger and diverse demographics of the women veteran population and evidence that women from these groups may experience disproportionate PTSD-related CVD risk.^14^^,^^19^^,^^20^ We examined these associations over a two-decade study period (2000–2019), spanning multiple service eras and secular changes in veteran demographics, trauma exposure, diagnostic criteria and practices, and cardiovascular care.

## Methods

### Data source

In this retrospective longitudinal study, we analyzed USA national VHA electronic health records (EHRs), including outpatient, inpatient, and purchased care (i.e., VHA-paid services provided by clinicians outside the VHA system). Patients were identified using the VHA Corporate Data Warehouse, a repository that incorporates patient-level data across the VHA national healthcare system.^21^

### Ethics statement

This study was approved by the University of California, Los Angeles Institutional Review Board (approval number IRB-18-0926). We followed the Strengthening the Reporting of Observational Studies in Epidemiology (STROBE) reporting guidelines^22^ in preparing this manuscript and adhered to the Declaration of Helsinki. As this was a retrospective database analysis and as thousands of the study subjects were diceased, obtaining consent was waived.

### Study cohort

**Fig. 1:**
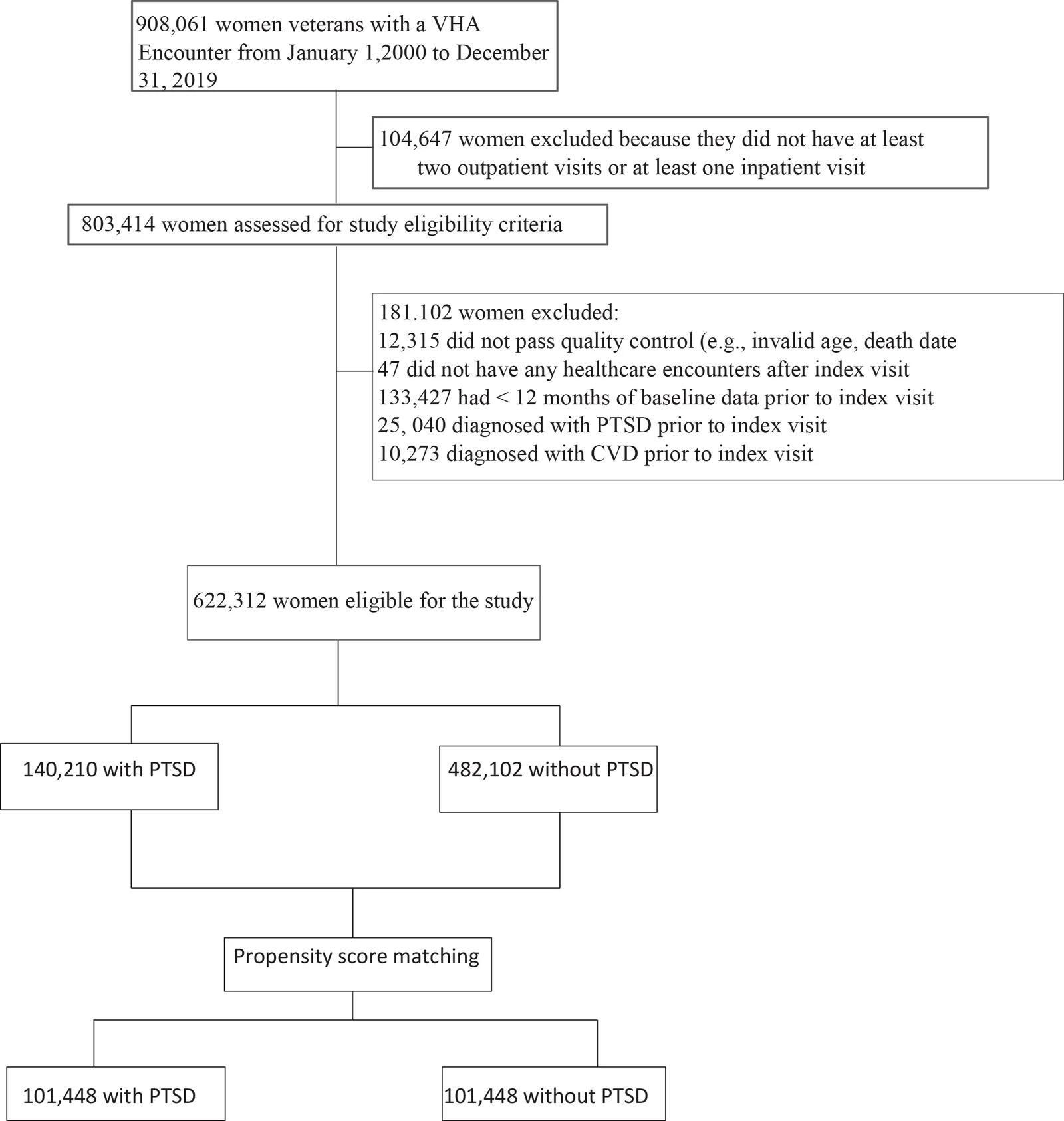
CONSORT diagram for creation of the study cohort.

We identified a cohort of women veterans aged 18 years or older who had a VHA healthcare encounter from 1/1/2000 to 12/31/2019. Women who did not pass data quality control (e.g., invalid death date or age), as well as those without any encounters after their initial healthcare encounter during the study period, were excluded. Any woman with a diagnosis of PTSD during the observation period was assigned to the PTSD group; the remaining were assigned to the control group. For women in the PTSD group, the first health encounter with a PTSD diagnosis served as their index visit, marking start of follow-up. For controls, the first visit occurring at least one year after the initial healthcare encounter served as their index visit. The 12 months before the index visit date served as a baseline period, whereby data on various CVD risk factors were assessed. We excluded women from the PTSD group with less than 12 months of baseline data; this did not apply to the control group, as all had sufficient baseline data. Any woman, whether in the PTSD or control group, who was diagnosed with any of the CVD conditions prior to or during this baseline period was also excluded. The remaining 622,312 women veterans comprised the pre-match sample (see Fig. 1 for exclusions).

### Variable definitions

#### Exposure

PTSD diagnosis (ever vs. never) was the exposure of interest. As in prior VHA EHR research,^19^^,^^20^^,^^23^, ^24^, ^25^, ^26^, ^27^, ^28^ PTSD was based on International Classification of Diseases, Ninth (ICD-9) or Tenth (ICD-10) Revision diagnosis codes (see Supplemental Table S1 for all variable definitions). We required PTSD documentation at one or more inpatient or two or more outpatient visits to indicate PTSD diagnostic status.^19^^,^^20^^,^^23^^,^^24^^,^^26^ Defining PTSD based on these EHR specifications has been found to be a valid approach, with one study reporting high sensitivity (0.99) and specificity (0.96).^24^

#### Outcomes

Our primary outcome was an incident CVD composite, reflecting first onset of the following diagnoses: IHD, stroke/TIA, HF, AF, PAD, PHTN, VTE, and AS. Similar to the approach for PTSD, each of the above cardiovascular conditions was defined by at least one inpatient or at least two outpatient documentations of ICD-9 or ICD-10 diagnosis codes. This approach increases the precision of identifying events in administrative data, particularly in outpatient records, and it has been used to define CVD outcomes (e.g., IHD, stroke/TIA, AF) in prior VHA EHR research.^9^^,^^19^^,^^20^^,^^29^ In addition to examining the CVD composite, we considered each of the above component conditions individually as secondary outcomes. Incident CVD was ascertained from index date to first event, death, or the end of the study period (12/31/2019).

#### Demographic and clinical characteristics

The following variables were balanced across PTSD exposures via propensity score matching to deter confounding: demographic factors (age, race/ethnicity), VHA healthcare engagement factors (year of index visit, number of healthcare records during the 12-month baseline), traditional CVD risk factors (hyperlipidemia, hypertension, diabetes, and smoking), female-specific risk factors (gestational hypertension and diabetes, placental disorders, preeclampsia), and other mental and physical health conditions (anxiety, depression, bipolar disorder, schizophrenia, alcohol use disorder, non-alcohol substance use disorder, obesity, chronic kidney disease, and neuroendocrine disorders) were extracted from the EHR. Age (continuous, plus grouped as 18–29, 30–39, 40–49, 50–59, 60+ for stratified analyses), and race/ethnicity (categorized as American Indian/Alaskan Native, Asian/Pacific Islander, Hispanic/Latina, non-Hispanic Black, non-Hispanic White, and other/unknown) were self-reported. Year of index visit along with the number of healthcare records with unique dates during the baseline 12-month period, were also extracted from the EHR. As previously described,^19^^,^^20^ ICD codes were used to define CVD risk factors using the same inpatient and outpatient validation criteria as for PTSD and CVD, with two exceptions. Obesity was defined by ICD codes or body mass index ≥30, calculated as weight (in kilograms) divided by mean height (in square meters).^23^ Smoking status was categorized as current or not current/unknown based on Health Factors data.^30^, ^31^, ^32^

### Statistical analysis

We used propensity score matching to create groups in which to estimate the association of PTSD with incident CVD among women veterans. By matching women without PTSD to those with PTSD, our analyses would lend insight to the association of PTSD with CVD among women with PTSD, analogous to the average treated effect among the treated (ATT). Propensity scores were estimated for all 622,312 women in the analytic sample (as specified in Fig. 1) using extreme gradient boosting (XGB), a tree-based machine learning algorithm that iteratively models residuals to improve fit. These kinds of machine learning techniques have been found to generate less biased estimates than standard parametric and semi-parametric techniques, as they are less vulnerable to misspecification (e.g., issues of overfitting) due to sample-splitting and cross-validation and cover a broader covariate space via interactions. To deter overfitting of the propensity model, we randomly divided the sample into training and test sets based on a 70/30 split. The training set was used to develop and tune the model, which included the aforementioned demographic and clinical characteristics as predictor variables, and the test set was used to evaluate the models' performance on new data. Ten-fold cross-validation was applied to the training set to derive optimal values of the XGB hyperparameters corresponding to eta, maximum tree depth, ratio of predictors subsampled, percentage of observations subsampled, and boosting iterations. Hyperparameters were optimized via grid search on accuracy of predictions. After determining the optimal model hyperparameters through the cross-validation process, the model was applied to the training and test sets, and Cohen's kappa agreement between observed and predicted values were calculated for each. Kappa values near 0 indicate no agreement, 0.41–0.60 moderate agreement, and 0.81–1.00 almost perfect agreement.^33^ In a final step, the XGB model was used to generate propensity scores representing predicted probabilities of PTSD status for the entire matched sample.

Nearest neighbor matching was performed on the XGB propensity scores, using a caliper of 0.25 and restricting matches to women veterans with no more than a 2-year age difference. Standardized mean differences (SMDs) were used to confirm covariate balance among women veterans with and without PTSD. To test the marginal association of PTSD with incident CVD, we conducted Cox survival models that accounted for the shared variance among matched pairs using the grouped jackknife method.^34^ The Fine–Gray method^35^ was utilized to account for the competing risk of death. This approach estimates subdistribution hazard ratios that describe differences in cumulative incidence in the presence of competing mortality and should be interpreted as descriptive measures rather than causal hazard effects.^36^ We assessed the proportional hazards assumption using tests of proportionality, where statistical significance indicates potential violation, and plots of Schoenfeld residuals. We first modeled time until diagnosis of the CVD composite outcome, with censoring applied to patients who were not diagnosed with CVD prior to the end of study period (12/31/19). We then modeled each of the component CVD outcomes separately. As a final step, we conducted age- and race/ethnicity-stratified analyses of the association of PTSD with the CVD composite.

Analyses were conducted using R, version 4.1.2. XGB and k-fold cross-validation were conducted using the ‘caret’ package for R, version 6.0-94. Nearest neighbor propensity score matching was performed using the ‘MatchIt’ package, version 4.5.3, and Cox models were conducted using the ‘survival’ package, version 3.5-5. We used a two-sided *p*-value <0.05 to indicate statistical significance.

### Role of the funding source

The funder of the study had no role in study design, data collection, data analysis, data interpretation, or writing of the report.

## Results

### Participant characteristics

Baseline characteristics and SMDs for the pre-match and matched samples of women veterans with and without PTSD are displayed in Table 1. In contrast to the pre-match sample, the matched sample achieved good balance across all covariates, with corresponding absolute values of SMDs <0.10. The final XGB model used to produce propensity scores for matching demonstrated moderate fit in both the training and test sets (Cohen's kappas for both = 0.51), indicating good generalizability of the model from the training to the test set. Number of healthcare records, year of index visit, depression, and anxiety conveyed the most importance in the propensity model, indicating that these variables explained key differences between women veterans with and without PTSD in the pre-match sample. As these variables also relate to CVD risk, we reduced the influence of these factors that could confound the PTSD-CVD association by accounting for these variables (and others) in the propensity score matching.

**Table 1:** Baseline demographics and clinical features of the pre-match and matched samples of women veterans with and without PTSD.

	Pre-match sample			Matched sample		
	No PTSD (*n* = 482,102)	PTSD (*n* = 140,210)	SMD	No PTSD (*n =* 101,448)	PTSD (*n =* 101,448)	SMD
Age (years)	43.08 (15.59)	40.10 (11.40)	−0.218	39.09 (11.53)	39.09 (11.51)	−0.001
Age group						
18–29	122,163 (25.3%)	33,327 (23.8%)	−0.036	28,877 (28.5%)	28,580 (28.2%)	−0.006
30–39	100,910 (20.9%)	40,684 (29.0%)	0.188	27,606 (27.2%)	28,069 (27.7%)	0.010
40–49	113,466 (23.5%)	35,271 (25.2%)	0.038	24,658 (24.3%)	24,732 (24.4%)	0.002
50–59	81,445 (16.9%)	24,362 (17.4%)	0.013	15,912 (15.7%)	15,713 (15.5%)	−0.005
60+	64,118 (13.3%)	6566 (4.7%)	−0.305	4395 (4.3%)	4354 (4.3%)	−0.002
Race/Ethnicity						
American Indian/Alaska Native	3549 (0.7%)	1749 (1.3%)	0.052	1135 (1.1%)	1254 (1.2%)	0.011
Asian/Pacific Islander	11,243 (2.3%)	3228 (2.3%)	−0.002	2692 (2.7%)	2448 (2.4%)	−0.015
Hispanic/Latina	31,248 (6.5%)	12,816 (9.1%)	0.099	9477 (9.3%)	9474 (9.3%)	−0.000
Non-Hispanic Black	114,765 (23.8%)	44,908 (32.0%)	0.184	30,278 (29.9%)	30,710 (30.3%)	0.009
Non-Hispanic White	244,347 (50.7%)	69,883 (49.8%)	−0.017	52,466 (51.7%)	51,340 (50.6%)	−0.022
Other/Unknown	76,950 (16.0%)	7626 (5.4%)	−0.345	5400 (5.3%)	6222 (6.1%)	0.035
Baseline conditions						
Hyperlipidemia	22,440 (4.7%)	22,235 (15.9%)	0.376	9411 (9.3%)	10,735 (10.6%)	0.044
Hypertension	38,013 (7.9%)	24,080 (17.2%)	0.283	11,613 (11.5%)	12,800 (12.6%)	0.036
Diabetes	9152 (1.9%)	7527 (5.4%)	0.186	2827 (2.8%)	3417 (3.4%)	0.034
Smoking	31,698 (6.6%)	40,309 (28.8%)	0.608	19,658 (19.4%)	22,096 (21.8%)	0.059
Obesity	61,016 (12.7%)	53,483 (38.1%)	0.612	28,469 (28.1%)	30,430 (30.0%)	0.043
Chronic kidney disease	648 (0.1%)	726 (0.5%)	0.067	237 (0.2%)	297 (0.3%)	0.012
Neuroendocrine disorders	12,666 (2.6%)	9621 (6.9%)	0.200	4367 (4.3%)	5045 (5.0%)	0.032
Female-specific risk factors	455 (0.1%)	1373 (1.0%)	0.121	323 (0.3%)	491 (0.5%)	0.026
Depression	7724 (1.6%)	33,289 (23.7%)	0.706	6782 (6.7%)	9367 (9.2%)	0.094
Bipolar disorder	3131 (0.7%)	8483 (6.1%)	0.304	2149 (2.1%)	3028 (3.0%)	0.055
Anxiety	6859 (1.4%)	25,827 (18.4%)	0.593	5791 (5.7%)	8015 (7.9%)	0.087
Schizophrenia	1224 (0.3%)	1178 (0.8%)	0.080	420 (0.4%)	543 (0.5%)	0.018
Alcohol use disorder	1280 (0.3%)	5280 (3.8%)	0.251	1086 (1.1%)	1686 (1.7%)	0.051
Drug use disorder	860 (0.2%)	4429 (3.2%)	0.234	749 (0.7%)	1341 (1.3%)	0.058
Number of baseline healthcare records	4.76 (9.2%)	17.11 (21.4%)	0.751	11.00 (14.1%)	12.04 (15.9%)	0.069
Year of index visit date						
2000	8 (0.0%)	0 (0.0%)	−0.006	1 (0.0%)	0 (0.0%)	−0.004
2001	48,371 (10.0%)	982 (0.7%)	−0.423	881 (0.9%)	974 (1.0%)	0.010
2002	29,230 (6.1%)	1517 (1.1%)	−0.271	1380 (1.4%)	1486 (1.5%)	0.009
2003	25,637 (5.3%)	1911 (1.4%)	−0.221	1621 (1.6%)	1850 (1.8%)	0.017
2004	23,800 (4.9%)	2755 (2.0%)	−0.163	2661 (2.6%)	2598 (2.6%)	−0.004
2005	21,919 (4.6%)	3416 (2.4%)	−0.115	3100 (3.1%)	3031 (3.0%)	−0.004
2006	20,892 (4.3%)	3643 (2.6%)	−0.095	3014 (3.0%)	3243 (3.2%)	0.013
2007	21,037 (4.4%)	4730 (3.4%)	−0.051	4257 (4.2%)	4046 (4.0%)	−0.010
2008	21,540 (4.5%)	5513 (3.9%)	−0.027	4925 (4.9%)	4596 (4.5%)	−0.015
2009	23,378 (4.9%)	6302 (4.5%)	−0.017	5114 (5.0%)	5091 (5.0%)	−0.001
2010	24,101 (5.0%)	6823 (4.9%)	−0.006	5671 (5.6%)	5505 (5.4%)	−0.007
2011	24,167 (5.0%)	7539 (5.4%)	0.016	6360 (6.3%)	6049 (6.0%)	−0.013
2012	25,136 (5.2%)	8513 (6.1%)	0.037	6777 (6.7%)	6698 (6.6%)	−0.003
2013	24,941 (5.2%)	9974 (7.1%)	0.081	7861 (7.8%)	7586 (7.5%)	−0.010
2014	25,244 (5.2%)	11,206 (8.0%)	0.111	8277 (8.2%)	8310 (8.2%)	0.001
2015	25,195 (5.2%)	12,733 (9.1%)	0.150	8629 (8.5%)	8953 (8.8%)	0.011
2016	27,824 (5.8%)	13,085 (9.3%)	0.135	7904 (7.8%)	8002 (7.9%)	0.004
2017	26,599 (5.5%)	12,978 (9.3%)	0.143	7698 (7.6%)	7809 (7.7%)	0.004
2018	24,018 (5.0%)	13,967 (10.0%)	0.190	7935 (7.8%)	8205 (8.1%)	0.010
2019	8 (0.0%)	0 (0.0%)	−0.006	1 (0.0%)	0 (0.0%)	−0.004

Means/frequencies and standard deviations/percentages (in parentheses) reported.

PTSD = posttraumatic stress disorder; SMD = standardized mean difference.

Women veterans in the analytic sample (*n* = 202,896) had a mean age of 39.1 years, with <5% of the study population aged 60 years or older. The population was diverse with respect to race/ethnicity as well (51.2% non-Hispanic White, 30.1% non-Hispanic Black, 9.3% Hispanic/Latina, 2.5% Asian/Pacific Islander, and 1.2% American Indian/Alaskan Native). Mean follow-up was 5.7 years (*SD* = 4.5) for women without PTSD and 6.2 years (*SD* = 4.4) for women with PTSD. During the follow-up period, 545 women without PTSD (0.5%) and 517 women with PTSD (0.5%) died.

### PTSD and incident CVD

**Fig. 2:**
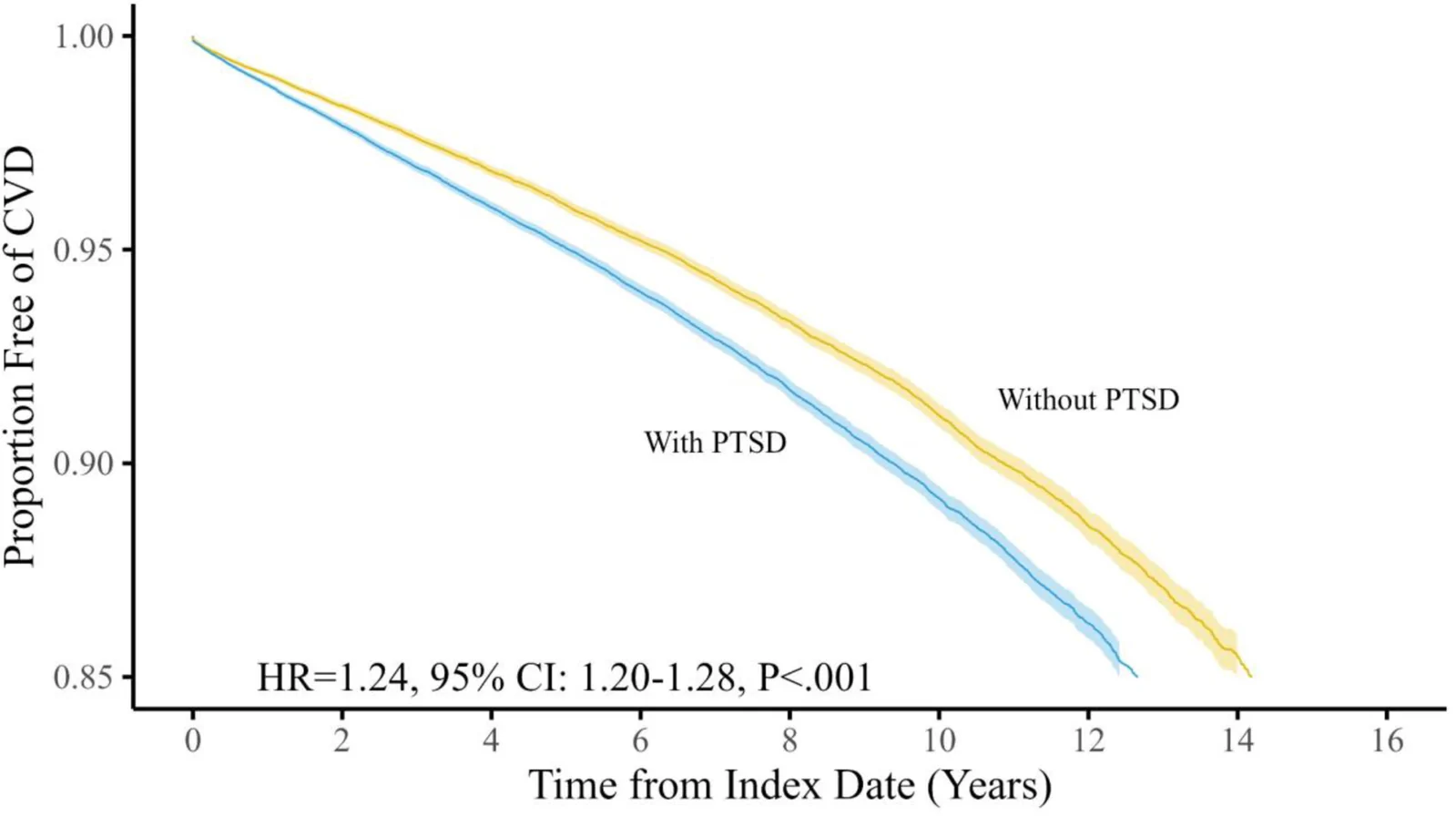
Kaplan–Meier survival curve comparing women veterans with and without posttraumatic stress disorder for the cardiovascular disease (CVD) composite outcome.

All Cox models satisfied the proportional hazards assumption according to tests of proportionality (*p*s ≥ 0.05) and inspection of Schoenfeld residual plots. Over the follow-up period, 5582 (5.5%) women without PTSD and 7562 (7.5%) women with PTSD developed one or more of the cardiovascular conditions used in the composite (Table 2). Kaplan–Meier survival curves for the CVD composite for women veterans with and without PTSD are depicted in Fig. 2. PTSD was significantly associated with an elevated rate of developing the incident CVD composite (HR = 1.24, 95% CI: 1.20–1.28; Table 2). In separate analyses of the component CVD diagnoses, PTSD was also significantly related to heightened incidence of IHD (HR = 1.30, 95% CI: 1.23–1.37), stroke/TIA (HR = 1.29, 95% CI: 1.21–1.38), HF (HR = 1.10, 95% CI: 1.02–1.18), PHTN (HR = 1.17, 95% CI: 1.02–1.35), VTE (HR = 1.34, 95% CI: 1.23–1.45), and AS (HR = 1.23, 95% CI: 1.03–1.46). Although the effect estimates were elevated for AF and PAD, these did not reach the level of statistical significance (Table 2).

**Table 2:** Associations of PTSD with incident CVD in women veterans.

Outcome	No PTSD	PTSD	Cox regression results	
	*n* (%) events	*n* (%) events	HR (95% CI)	*p*-value
CVD Composite	5582 (5.5%)	7562 (7.5%)	1.24 (1.20–1.28)	<0.001
IHD	2130 (2.1%)	3066 (3.0%)	1.30 (1.23–1.37)	<0.001
Stroke/TIA	1543 (1.5%)	2207 (2.2%)	1.29 (1.21–1.38)	<0.001
HF	1256 (1.2%)	1544 (1.5%)	1.10 (1.02–1.18)	0.012
AF	699 (0.7%)	840 (0.8%)	1.07 (0.97–1.18)	0.16
PAD	583 (0.6%)	689 (0.7%)	1.06 (0.95–1.18)	0.33
PHTN	338 (0.3%)	443 (0.4%)	1.17 (1.02–1.35)	0.029
VTE	956 (0.5%)	1415 (0.7%)	1.34 (1.23–1.45)	<0.001
AS	223 (0.2%)	307 (0.3%)	1.23 (1.03–1.46)	0.019

AF = atrial fibrillation/flutter; AS = aortic valve stenosis; CI = confidence interval; CVD = cardiovascular disease; HF = heart failure/cardiomyopathy; HR = hazard ratio; IHD = ischemic heart disease; PAD = peripheral arterial disease; PHTN = pulmonary arterial hypertension/pulmonary hypertension; PTSD = posttraumatic stress disorder; TIA = transient ischemic attack; VTE = venous thromboembolism.

### Stratified analyses of PTSD and CVD: age and race/ethnicity

The results of analyses stratified by age and race/ethnicity are reported in Table 3. PTSD was associated with elevated rates of the incident CVD composite outcome for all age groups, with particularly strong associations for women under 40 years. In analyses stratified by race/ethnicity, PTSD was related to significantly elevated rates of developing CVD for all groups except women of Asian/Pacific Islander descent, for whom there was a trend towards an association of PTSD with greater CVD incidence (HR = 1.34; 95% CI: 0.99–1.82). The PTSD- CVD relation was especially strong for American Indian/Alaska Native (HR = 1.58; 95% CI: 1.11–2.26) and Hispanic/Latina women (HR = 1.50; 95% CI: 1.27–1.77). We note that sample sizes for some racial and ethnic groups (e.g., Asian/Pacific Islander, American Indian/Alaska Native) were small, and confidence intervals were wide, suggesting a lack of precision in estimates.

**Table 3:** Age- and race/ethnicity-stratified analyses of association of PTSD with incident CVD in women veterans.

	*n*	No PTSD	PTSD	Cox regression results	
		*n* (%) events	*n* (%) events	HR (95% CI)	*p*-value
Age					
18–29	57,457	368 (1.3%)	590 (2.1%)	1.42 (1.24–1.61)	<0.001
30–39	55,675	720 (2.6%)	1213 (4.3%)	1.52 (1.39–1.67)	<0.001
40–49	49,390	1776 (7.2%)	2501 (10.1%)	1.31 (1.23–1.39)	<0.001
50–59	31,625	1981 (12.4%)	2332 (14.8%)	1.13 (1.06–1.19)	<0.001
60+	8749	737 (16.8%)	926 (21.3%)	1.17 (1.07–1.29)	<0.001
Race/Ethnicity					
American Indian/Alaska Native	2389	44 (3.9%)	91 (7.3%)	1.58 (1.11–2.26)	0.012
Asian/Pacific Islander	5140	71 (2.6%)	104 (4.3%)	1.34 (0.99–1.82)	0.057
Hispanic/Latina	18,951	221 (2.3%)	367 (3.9%)	1.50 (1.27–1.77)	<0.001
Non-Hispanic Black	60,988	1766 (5.8%)	2157 (7.0%)	1.13 (1.06–1.21)	<0.001
Non-Hispanic White	103,806	3268 (6.2%)	4448 (8.7%)	1.27 (1.22–1.33)	<0.001
Other/Unknown	11,622	212 (3.9%)	395 (6.3%)	1.31 (1.11–1.54)	0.002

CI = confidence interval; CVD = cardiovascular disease; HR = hazard ratio; PTSD = posttraumatic stress disorder.

## Discussion

Mental health conditions such as PTSD are increasingly being recognized as nontraditional CVD risk factors,^3^ with a recent Scientific Statement from the American Heart Association highlighting the importance of the mind-heart-body connection.^37^ However, much of the literature on the PTSD-CVD relation is based on men, and these studies have predominantly investigated IHD or stroke.^4^^,^^5^ Women veterans have been notably underrepresented in this research, despite having substantially higher levels of traditional (e.g., hypertension, hyperlipidemia) and nontraditional (e.g., trauma, PTSD) CVD risk factors than civilian women and men veterans.^14^ The distinct demographics of this growing group of veterans further warrants research specifically in this population.^15^ In a study of over 200,000 women veterans spanning two decades, we addressed this limitation by investigating the association of PTSD with a wide range of incident CVD outcomes. In groups matched on various CVD risk factors that could potentially confound the PTSD-CVD relation, we found that PTSD was significantly associated with elevated rates of developing an incident CVD composite, comprising IHD, stroke/TIA, HF, AF, PAD, PHTN, VTE, and AS. Indeed, in this relatively young cohort of women veterans, incidence of the CVD composite was 7.5% vs. 5.5% in those with vs. without PTSD—a 36% relative increase based on crude cumulative incidence. In analyses of separate cardiovascular conditions, PTSD was significantly associated with greater incidence of IHD, stroke/TIA, HF, PHTN, VTE, and AS. Moreover, the link between PTSD and incident CVD was most pronounced in women veterans younger than 40 years of age and in American Indian/Alaska Native and Hispanic/Latina women veterans.

The results of our study suggest that the cardiovascular consequences of PTSD are wide-ranging, and they mirror findings from a large population-based health registry study in Sweden linking PTSD with incident hypertensive disease, IHD, cerebrovascular disease, emboli/thrombosis, and HF.^11^ Furthermore, to the best of our knowledge, we document the first associations between PTSD with PHTN and AS, and between PTSD and HF specifically in women veterans. However, in contrast to research in predominantly male veteran samples,^9^^,^^10^ we did not detect significant associations between PTSD and incident AF or PAD (when examined as individual outcomes), although the effect estimates were elevated. Several of the cardiovascular conditions were rare in this cohort of younger women with an average follow-up of approximately 6 years, which may have limited our ability to detect statistically significant associations with PTSD. Indeed, considering the young mean age at baseline (39.1 years) and average follow-up time, our results reflects an overall early risk window for CVD, which may limit generalizability to older women and to long-term CVD incidence. Further research in larger samples of men and women veterans with longer follow-up is needed; such research can also assess whether there may be any potential sex differences in PTSD-CVD relations. However, to date, most studies have documented generally similar associations between PTSD and cardiovascular outcomes in men and women.^6^^,^^9^

Numerous behavioral and physiological mechanisms may underlie the link between PTSD and these incident CVD outcomes.^38^ For example, PTSD has been linked to a variety of unhealthy behaviors (e.g., physical inactivity, poor diet, and disrupted sleep) and comorbid mental health conditions (e.g., depression, substance use disorders) that may contribute to CVD risk.^2^^,^^39^^,^^40^ In addition, dysfunctional engagement of stress response systems in PTSD (e.g., dysregulation of the hypothalamic-pituitary-adrenal axis, hyperreactivity of the sympathetic nervous system)—particularly in response to trauma-related reminders—may have adverse downstream consequences for cardiovascular health (e.g., vasoconstriction, endothelial dysfunction, arterial stiffness, and cardiac afterload), leading to IHD or stroke.^2^^,^^38^^,^^41^ Immune dysregulation and chronic systemic inflammation may also underlie associations of PTSD with various CVD outcomes, such as HF or PHTN.^42^ Research on mechanisms linking PTSD with incident CVD been limited in women veterans. In one exception, we used VHA EHR data to demonstrate that a range of traditional and nontraditional CVD risk factors together explained less than 50% of the association of PTSD with incident IHD in women veterans, although both traditional risk factors and mental health conditions were prominent pathway variables.^43^ These findings highlight the importance of further research to identify additional modifiable CVD risk factors in women veterans with PTSD, including studying key lifestyle factors such as sleep patterns and physical activity and trauma-related processes not captured in EHR data. Such mechanism-focused research has the potential to improve our understanding of the PTSD-CVD association.

Understanding the potential impact of PTSD on a broad set of CVD outcomes has important clinical implications, particularly for younger women. Indeed, we observed some of the strongest associations between PTSD and greater incidence of the CVD composite in women aged 18–40 years. This pattern of results mirrors prior research linking PTSD to early onset of CVD,^6^^,^^19^^,^^20^^,^^44^^,^^45^ and it suggests that CVD risk screening may be warranted at even younger ages in women—particularly for those with PTSD and women veterans. Historically, leading cardiology risk assessment guidelines have categorized women younger than age 45 as being at low risk for CVD events,^46^ and the most recent Veterans Affairs/Department of Defense Clinical Practice Guidelines for the management of dyslipidemia recommend screening at age 40 and older, with no mention of PTSD status.^47^ However, research conducted specifically in women veterans found that 10-year atherosclerotic CVD risk increased steadily from age 30 onward in Hispanic/Latina and non-Hispanic Black and White women veterans,^48^ suggesting an acceleration of the aging trajectory of CVD risk and a need to assess risk earlier and in more tailored ways in this population.^49^ Notably, there have been recent efforts in the VHA to develop and implement a Cardiovascular Toolkit for women veterans that aims to improve patient education, CVD risk identification and documentation, and engagement in services,^18^ which may hold promise.

Although PTSD was associated with greater incidence of CVD in women veterans of various racial and ethnic backgrounds, these links were especially pronounced for American Indian/Alaska Native and Hispanic/Latina women. Nevertheless, interpretation of estimates for some sub-groups is limited by small sample sizes and wide confidence intervals, and additional research in larger samples is needed. At the same time, racial and ethnic disparities in PTSD^50^ and CVD^51^ are well-documented, and social determinants of health may play a significant role in these disparities. Although our findings highlight some groups for whom the PTSD-CVD link may be particularly strong and who thus might benefit from focused screening, ultimately providing equitable access to healthcare and CVD prevention efforts to all women veterans is critical.

The current study is subject to several limitations characterizing retrospective analyses of administrative diagnostic data, including misclassification, misestimation of onset time, ascertainment bias, and issues with representativeness. Additionally, although we comprehensively accounted for a number of risk factors in propensity score matching, not all potential risk factors, such as menopausal status, diet, physical activity, or sleep, could be assessed due to limitations of the information available in EHRs. Despite also accounting for several mental health conditions in our approach (anxiety, depression, bipolar disorder, schizophrenia, and substance use disorders), we did not consider personality-related factors and disorders (e.g., neuroticism, Type D personality, and borderline personality disorder), which have been linked to CVD risk.^52^, ^53^, ^54^ Furthermore, we lacked other relevant information about trauma and related psychopathology, including traumatic experiences in childhood and adulthood, PTSD symptom severity, and receipt of evidence-based psychological interventions for PTSD. Additionally, smoking was coded as current vs. not current/unknown, which combines former and never smokers and may misclassify risk. Overall, although an extensive array of clinical covariates was included in propensity score models, residual confounding by unmeasured social and behavioral factors is also likely. Furthermore, the study period of 2000–2019 spans multiple service eras and secular changes in veteran demographics, trauma exposure, diagnostic criteria and practices, and cardiovascular care. For example, PTSD diagnostic criteria changed from DSM IV to DSM 5 over the course of the study period, and the ICD diagnostic codes reflect slightly different constructs. Although we matched women veterans with and without PTSD on year of index visit, our findings merit consideration in light of these cohort-related factors. Finally, our results are based on a large cohort of women veteran users of the VHA and may not be generalizable to other women veterans, other women, and men. Nevertheless, our study also had many strengths. This is the largest investigation of the association of PTSD with a comprehensive set of CVD outcomes in women veterans, analyzing data from over 200,000 women spanning two decades. In addition, we matched women veterans with and without PTSD on a comprehensive list of potential risk factors beyond the traditional CVD risk factors, including female-specific risk factors and a range of mental and physical health conditions. Furthermore, because of the large sample size, we were able to perform stratified analysis based on age and race/ethnicity, thus uncovering subgroups with particularly elevated risk.

In conclusion, our study suggests that PTSD is associated with heightened incidence of a broad range of CVD outcomes in women veterans, including PHTN and AS—outcomes never reported before based on our review of the PTSD-CVD literature. Further research is warranted to elucidate underlying mechanisms and to evaluate the impact of tailored interventions aimed at reducing cardiovascular risk in this high-risk population.

## Contributors

RE, PAD, and JAS conceived the study. RE, PAD, CAA, ALS, and JCB designed the methodology and analytic plan. PAD and CAA performed the statistical analyses. RE and JAS drafted the initial manuscript. All authors contributed to data interpretation, revised the manuscript critically for important intellectual content, and approved the final version. RE is the guarantor and accepts full responsibility for the integrity of the work, the decision to publish, and the overall content. CAA and PAD accessed and verified the data.

## Data sharing statement

The data underlying this study are from the United States Veterans Health Administration (VHA) and are not publicly available due to privacy and regulatory restrictions. Researchers who meet the criteria for access to these confidential data may apply through the VHA data request processes.

## Declaration of interests

RE and JAS report grants from the US National Institutes of Health/National Heart, Lung, and Blood Institute. PAD reports grants from the US Advanced Research Projects Agency for Health, National Institute on Deafness and Other Communication Disorders, and National Institute on Mental Health. CAA reports research grants from Boehringer Ingelheim, Bristol Myers Squibb, and Pfizer; and payment for expert testimony from Glast Phillips Murray Zopolsky. ALS and JCB declare no competing interests.
