# Supplementary material for: Impact of posttraumatic stress disorder on incident cardiovascular outcomes in women veterans: a retrospective longitudinal study
Source: eClinicalMedicine. 2026 Sep 8;100:104197. doi: 10.1016/j.eclinm.2026.104197 (PMC13579694; doi:10.1016/j.eclinm.2026.104197)
Supplement: Supplementary file 1 [file mmc1.docx]

**Supplemental Table 1.** Variable definitions.

| Variable | Definition |
| --- | --- |
| ***Exposure*** | Record of at least 2 outpatient or 1 inpatient diagnosis codes between 1/1/2000-12/31/2019 |
| PTSD | ICD-9: 309.81  ICD-10: F43.1, F43.10, F43.11, F43.12 |
| ***Outcome*** | Record of at least 2 outpatient or 1 inpatient diagnosis code |
| Aortic valve stenosis | ICD-9: 424.1  ICD-10: I35.0, I35.1, I35.2 |
| Atrial fibrillation/flutter | ICD-9: 427.31, 427.32  ICD-10: I48.91, I48.92 |
| Heart failure/cardiomyopathy | ICD-9: 398.91, 402.01, 402.11, 402.91, 404.01, 404.03, 404.11, 404.13, 404.91, 404.93, 425, 425.8, 428, 428.22, 428.3, 428.9  ICD-10: I11.0, I13.0, I13.2, I25.5, I42.0, I42.5, I42.6, I42.7, I42.8, I42.9, I43, I50., I50.20, I50.30, I50.9 |
| Ischemic heart disease | ICD-9: 410.01, 410.02, 410.1, 410.11, 410.12, 410.21, 410.22, 410.31, 410.32, 410.41, 410.42, 410.51, 410.52, 410.61, 410.62, 410.71, 410.72, 410.81, 410.82, 410.9, 410.91, 410.92, 412, 413.1, 413.9, 414.01, 414.02, 414.03, 414.04, 414.05, 414.06, 414.07, 429.79  ICD-10: I20.0, I20.1, I20.8, I20.9, I21.01, I21.02, I21.09, I21.11, I21.19, I21.21, I21.29, I21.3, I21.4, I21.9, I21.A1, I21.A9, I22.0, I22.1, I22.2, I22.8, I22.9, I23.1, I23.2, I23.3, I23.4, I23.6, I23.7, I23.8, I25.10, I25.110, I25.111, I25.118, I25.119 |
| Peripheral arterial disease | ICD-10: I70.201-209, I70.211-219, I70.221-229, I70.231-261-269 |
| Pulmonary arterial hypertension/pulmonary hypertension | ICD-10: I27.0, I27.2, I27.89  ICD 9:416.0, 416.8, 416.9 |
| Stroke/transient ischemic attack | ICD-9: 362.3, 362.30, 362.31, 362.32, 362.33, 362.34, 362.35, 362.36, 362.37, 430, 431, 432, 432.0, 432.1, 432.9, 433.01, 433.11, 433.21, 433.31, 433.81, 433.91, 434, 434.00, 434.01, 434.1, 434.10, 434.11, 434.9, 434.90, 434.91, 435, 435.0, 435.1, 435.2, 435.3, 435.8, 435.9, 436  ICD-10: G45, G45.0, G45.1, G45.2, G45.3, G45.4, G45.8, G45.9, H34.1, H34.10, H34.11, H34.12, H34.13, I60, I60.0, I60.00, I60.01, I60.02, I60.1, I60.10, I60.11, I60.12, I60.2, I60.20, I60.21, I60.22, I60.3, I60.30, I60.31, I60.32, I60.4, I60.5, I60.50, I60.51, I60.52, I60.6, I60.7, I60.8, I60.9, I61, I61.0, I61.1, I61.2, I61.3, I61.4, I61.5, I61.6, I61.8, I61.9, I63, I63.0, I63.00, I63.01, I63.011, I63.012, I63.013, I63.019, I63.02, I63.03, I63.031, I63.032, I63.033, I63.039, I63.09, I63.1, I63.10, I63.11, I63.111, I63.112, I63.113, I63.119, I63.12, I63.13, I63.131, I63.132, I63.133, I63.139, I63.19, I63.2, I63.20, I63.21, I63.211, I63.212, I63.213, I63.219, I63.22, I63.23, I63.231, I63.232, I63.233, I63.239, I63.29, I63.3, I63.30, I63.31, I63.311, I63.312, I63.313, I63.319, I63.32, I63.321, I63.322, I63.323, I63.329, I63.33, I63.331, I63.332, I63.333, I63.339, I63.34, I63.341, I63.342, I63.343, I63.349, I63.39, I63.4, I63.40, I63.41, I63.411, I63.412, I63.413, I63.419, I63.42, I63.421, I63.422, I63.423, I63.429, I63.43, I63.431, I63.432, I63.433, I63.439, I63.44, I63.441, I63.442, I63.443, I63.449, I63.49, I63.5, I63.50, I63.51, I63.511, I63.512, I63.513, I63.519, I63.52, I63.521, I63.522, I63.523, I63.529, I63.53, I63.531, I63.532, I63.533, I63.539, I63.54, I63.541, I63.542, I63.543, I63.549, I63.59, I63.6, I63.8, I63.81, I63.89, I63.9, I67.89 |
| Venous thromboembolism | ICD-9: 415.1, 427.1, 427.41, 451, 451.1, 451.2, 451.81, 451.83, 451.84, 451.89, 451.9, 452, 453, 453.0, 453.1, 453.2, 453.3, 453.4, 453.40, 453.5, 453.50, 453.72, 453.73, 453.74, 453.75, 453.76, 453.77, 453.79, 453.8, 453.82, 453.83, 453.84, 453.85, 453.86, 453.87, 453.89, 453.9  ICD-10: I26.9, I82. 40, I82.409, I82.509, I82.599 |
| ***Demographics*** |  |
| Age at index visit, years | Date difference (day, Date of Birth, Index date)/365.25 |
| Race/ethnicity | American Indian/Alaska Native, Asian/Pacific Islander, Hispanic/Latina, non-Hispanic Black, non-Hispanic White, other/unknown |
| ***VHA Healthcare Engagement Factors*** |  |
| Year of index visit | Year of 1) the first visit with a PTSD diagnosis for women with PTSD or 2) the first visit occurring at least one year after the initial healthcare encounter for women without PTSD |
| Number of baseline healthcare records | Number of unique dates in which a new record/entry was made during the 12-month baseline |
| ***Traditional CVD Risk Factors*** |  |
| Hyperlipidemia | ICD9: 272.2, 272.4  ICD10: E78.5 |
| Hypertension | ICD9: 402.11, 403.9, 405.91, 401.1, 402.91, 404.9, 404.91, 404.93, 403.91, 404.01, 404.02, 404.13, 405.09, 405.11, 405.19, 405.99, 401.9, 402.01, 403.01, 403.11, 404.03, 404.11, 404.12, 404.92, 405.01  ICD10: I10, I11.0, I11.9, I15.0, I15.1, I15.2, I15.8, I15.9, I16.0, I16.1, I16.9 |
| Diabetes | ICD9: 250, 250.01, 250.02, 250.03, 250.1, 250.11, 250.12, 250.13, 250.2, 250.21, 250.22, 250.23, 250.3, 250.31, 250.32, 250.33, 250.4, 250.41, 250.42, 250.43, 250.5, 250.51, 250.52, 250.53, 250.6, 250.61, 250.62, 250.63, 250.7, 250.71, 250.72, 250.73, 250.8, 250.81, 250.82, 250.83, 250.9, 250.91, 250.92, 250.93, 357.2  ICD10: E08.00, E08.01, E08.10, E08.11, E08.21, E08.22, E08.29, E08.311, E08.319, E08.321, E08.3211, E08.3212, E08.3213, E08.3219, E08.329, E08.3291, E08.3292, E08.3293, E08.3299, E08.331, E08.3311, E08.3312, E08.3313, E08.3319, E08.339, E08.3391, E08.3392, E08.3393, E08.3399, E08.341, E08.3411, E08.3412, E08.3413, E08.3419, E08.349, E08.3491, E08.3492, E08.3493, E08.3499, E08.351, E08.3511, E08.3512, E08.3513, E08.3519, E08.3521, E08.3522, E08.3523, E08.3529, E08.3531, E08.3532, E08.3533, E08.3539, E08.3541, E08.3542, E08.3543, E08.3549, E08.3551, E08.3552, E08.3553, E08.3559, E08.359, E08.3591, E08.3592, E08.3593, E08.3599, E08.36, E08.37X1, E08.37X2, E08.37X3, E08.37X9, E08.39, E08.40, E08.41, E08.42, E08.43, E08.44, E08.49, E08.51, E08.52, E08.59, E08.610, E08.618, E08.620, E08.621, E08.622, E08.628, E08.630, E08.638, E08.641, E08.649, E08.65, E08.69, E08.8, E08.9, E09.00, E09.01, E09.10, E09.11, E09.21, E09.22, E09.29, E09.311, E09.319, E09.321, E09.3211, E09.3212, E09.3213, E09.3219, E09.329, E09.3291, E09.3292, E09.3293, E09.3299, E09.331, E09.3311, E09.3312, E09.3313, E09.3319, E09.339, E09.3391, E09.3392, E09.3393, E09.3399, E09.341, E09.3411, E09.3412, E09.3413, E09.3419, E09.349, E09.3491, E09.3492, E09.3493, E09.3499, E09.351, E09.3511, E09.3512, E09.3513, E09.3519, E09.3521, E09.3522, E09.3523, E09.3529, E09.3531, E09.3532, E09.3533, E09.3539, E09.3541, E09.3542, E09.3543, E09.3549, E09.3551, E09.3552, E09.3553, E09.3559, E09.359, E09.3591, E09.3592, E09.3593, E09.3599, E09.36, E09.37X1, E09.37X2, E09.37X3, E09.37X9, E09.39, E09.40, E09.41, E09.42, E09.43, E09.44, E09.49, E09.51, E09.52, E09.59, E09.610, E09.618, E09.620, E09.621, E09.622, E09.628, E09.630, E09.638, E09.641, E09.649, E09.65, E09.69, E09.8, E09.9, E10.10, E10.11, E10.21, E10.22, E10.29, E10.311, E10.319, E10.321, E10.3211, E10.3212, E10.3213, E10.3219, E10.329, E10.3291, E10.3292, E10.3293, E10.3299, E10.331, E10.3311, E10.3312, E10.3313, E10.3319, E10.339, E10.3391, E10.3392, E10.3393, E10.3399, E10.341, E10.3411, E10.3412, E10.3413, E10.3419, E10.349, E10.3491, E10.3492, E10.3493, E10.3499, E10.351, E10.3511, E10.3512, E10.3513, E10.3519, E10.3521, E10.3522, E10.3523, E10.3529, E10.3531, E10.3532, E10.3533, E10.3539, E10.3541, E10.3542, E10.3543, E10.3549, E10.3551, E10.3552, E10.3553, E10.3559, E10.359, E10.3591, E10.3592, E10.3593, E10.3599, E10.36, E10.37X1, E10.37X2, E10.37X3, E10.37X9, E10.39, E10.40, E10.41, E10.42, E10.43, E10.44, E10.49, E10.51, E10.52, E10.59, E10.610, E10.618, E10.620, E10.621, E10.622, E10.628, E10.630, E10.638, E10.641, E10.649, E10.65, E10.69, E10.8, E10.9, E11.00, E11.01, E11.10, E11.11, E11.21, E11.22, E11.29, E11.311, E11.319, E11.321, E11.3211, E11.3212, E11.3213, E11.3219, E11.329, E11.3291, E11.3292, E11.3293, E11.3299, E11.331, E11.3311, E11.3312, E11.3313, E11.3319, E11.339, E11.3391, E11.3392, E11.3393, E11.3399, E11.341, E11.3411, E11.3412, E11.3413, E11.3419, E11.349, E11.3491, E11.3492, E11.3493, E11.3499, E11.351, E11.3511, E11.3512, E11.3513, E11.3519, E11.3521, E11.3522, E11.3523, E11.3529, E11.3531, E11.3532, E11.3533, E11.3539, E11.3541, E11.3542, E11.3543, E11.3549, E11.3551, E11.3552, E11.3553, E11.3559, E11.359, E11.3591, E11.3592, E11.3593, E11.3599, E11.36, E11.37X1, E11.37X2, E11.37X3, E11.37X9, E11.39, E11.40, E11.41, E11.42, E11.43, E11.44, E11.49, E11.51, E11.52, E11.59, E11.610, E11.618, E11.620, E11.621, E11.622, E11.628, E11.630, E11.638, E11.641, E11.649, E11.65, E11.69, E11.8, E11.9, E13.00, E13.01, E13.10, E13.11, E13.21, E13.22, E13.29, E13.311, E13.319, E13.321, E13.3211, E13.3212, E13.3213, E13.3219, E13.329, E13.3291, E13.3292, E13.3293, E13.3299, E13.331, E13.3311, E13.3312, E13.3313, E13.3319, E13.339, E13.3391, E13.3392, E13.3393, E13.3399, E13.341, E13.3411, E13.3412, E13.3413, E13.3419, E13.349, E13.3491, E13.3492, E13.3493, E13.3499, E13.351, E13.3511, E13.3512, E13.3513, E13.3519, E13.3521, E13.3522, E13.3523, E13.3529, E13.3531, E13.3532, E13.3533, E13.3539, E13.3541, E13.3542, E13.3543, E13.3549, E13.3551, E13.3552, E13.3553, E13.3559, E13.359, E13.3591, E13.3592, E13.3593, E13.3599, E13.36, E13.37X1, E13.37X2, E13.37X3, E13.37X9, E13.39, E13.40, E13.41, E13.42, E13.43, E13.44, E13.49, E13.51, E13.52, E13.59, E13.610, E13.618, E13.620, E13.621, E13.622, E13.628, E13.630, E13.638, E13.641, E13.649, E13.65, E13.69, E13.8, E13.9, 253.5, E23.2, N25.1 |
| Smoking | ICD9: 305.1, 649.00, 649.01, 649.02, 649.03, 649.04, F17.200,  ICD10: F17.201, F17.203, F17.208, F17.209, F17.210, F17.211, Z87.891  VA Health Factors |
| ***Female-Specific Risk Factors^a^*** | ICD-9: 641.2, 641.21, 641.23, 642.3, 642.31, 642.32, 642.33, 642.4, 642.41, 642.42, 642.43, 642.44, 642.5, 642.51, 642.52, 642.53, 642.54, 642.6, 642.61, 642.62, 642.63, 642.64, 642.70, 642.71, 642.72, 642.73, 642.74, 642.9, 642.91, 642.92, 642.93, 648.80, 648.81, 648.82, 648.83, 648.84, 656.7, 656.71, 656.73  ICD-10: O13.1, O13.2, O13.3, O13.4, O13.5, O13.9, O14.00, O14.02, O14.03, O14.04, O14.05, O14.10, O14.12, O14.13, O14.14, O14.15, O14.20, O14.22, O14.23, O14.24, O14.25, O14.90, O14.92, O14.93, O14.94, O14.95, O16.1, O16.2, O16.3, O16.4, O16.5, O16.9, O24.410, O24.414, O24.415, O24.419, O24.420, O24.424, O24.425, O24.429, O24.430, O24.434, O24.435, O24.439, O43.811, O43.812, O43.813, O43.819, O45.001, O45.002, O45.003, O45.009, O45.011, O45.012, O45.013, O45.019, O45.021, O45.022, O45.023, O45.029, O45.091, O45.092, O45.093, O45.099, O45.8X1, O45.8X1, O45.8X2, O45.8X2, O45.8X3, O45.8X3, O45.8X9, O45.8X9, O45.90, O45.91, O45.92, O45.93, Z86.32 |
| ***Other Mental and Physical Health Conditions*** |  |
| Chronic kidney disease | ICD-9: 585, 585.1, 585.2, 585.3, 585.4, 585.5, 585.6, 585.9, 586, 587, 588, 588.1, 588.81, 588.89, 588.9  ICD-10: I12.0, I12.9, I13.0, I13.10, I13.11, I13.2, E08.22, E09.22, E10.22, E11.22, E13.22, N18, N18.1, N18.2, N18.3, N18.4, N18.5, N18.6, N18.9 |
| Neuroendocrine disorders^b^ | ICD-9: 194, 198.7, 227.3, 242.4, 242.41, 242.8, 242.81, 242.9, 242.91, 243, 244, 244.1, 244.2, 244.3, 244.8, 244.9, 253.3, 253.5, 255, 281  ICD-10: C74.00, C74.01, C74.02, C74.02, C74.10, C74.11, C74.12, C74.90, C74.91, C74.91, C74.92, C79.70, C70.71, C79.72, D35.2, E03.0, E03.1, E03.2, E03.3, E03.4, E03.5, E03.8, E03.9, E05.00, E05.01, E05.10, E05.11, E05.20, E05.21, E05.30, E05.31, E05.40, E05.41, E05.80, E05.81, E05.90, E05.91, E23.0, E23.2, E24.0, E24.2, E24.4, E24.8, E24.9, E27.1, N25.1, P72.1 |
| Obesity | ICD-9: 278.01, 278.00  ICD-10: O99.215, O99.214, O99.213, O99.212, O99.211, O99.210, E66.9, E66.8, E66.3, E66.2, E66.1, E66.09, E66.01  BMI ≥30 k/m^2^ |
| Depression | ICD-9: 296.2, 296.21, 296.22, 296.23, 296.24, 296.25, 296.26, 296.3, 296.31, 296.32, 296.33, 296.34, 296.35, 296.36  ICD-10: F32.0, F32.1, F32.2, F32.3, F32.4, F32.5, F32.9, F33.0, F33.1, F33.2, F33.3, F33.40, F33.41, F33.42, F33.8, F33.9 |
| Anxiety | ICD-9: 300.01, 300.02, 300.09  ICD-10: F41.0, F41.1, F41.3, F41.8, F41.9 |
| Bipolar disorder | ICD-9: 296, 296.01, 296.02, 296.03, 296.04, 296.05, 296.06, 296.1, 296.11, 296.12, 296.13, 296.14, 296.15, 296.16, 296.2, 296.21, 296.22, 296.23, 296.24, 296.25, 296.26, 296.3, 296.31, 296.32, 296.33, 296.34, 296.35, 296.36, 296.4, 296.41, 296.42, 296.43, 296.44, 296.45, 296.46, 296.5, 296.51, 296.52, 296.53, 296.54, 296.55, 296.56, 296.6, 296.61, 296.62, 296.63, 296.64, 296.65, 296.66, 296.7, 296.8, 296.81, 296.82, 296.89, 296.9, 296.99  ICD-10: F31., F31.0, F31.1, F31.10, F31.11, F31.12, F31.13, F31.2, F31.3, F31.30, F31.31, F31.32, F31.4, F31.5, F31.6, F31.60, F31.61, F31.62, F31.63, F31.64, F31.7, F31.70, F31.71, F31.72, F31.73, F31.74, F31.75, F31.76, F31.77, F31.78, F31.8, F31.81, F31.89, F31.9 |
| Schizophrenia | ICD-9: 295, 295.01, 295.02, 295.03, 295.04, 295.05, 295.10, 295.11, 295.12, 295.13, 295.14, 295.15, 295.2, 295.21, 295.22, 295.23, 295.24, 295.25, 295.3, 295.31, 295.32, 295.33, 295.34, 295.35, 295.40, 295.41, 295.42, 295.43, 295.44, 295.45, 295.5, 295.51, 295.52, 295.53, 295.54, 295.55, 295.60, 295.61, 295.62, 295.63, 295.64, 295.65, 295.7, 295.71, 295.72, 295.73, 295.74, 295.75, 295.8, 295.81, 295.82, 295.83, 295.84, 295.85, 295.90, 295.91, 295.92, 295.93, 295.94, 295.95  ICD-10: F20.0, F20.1, F20.2, F20.3, F20.5, F20.81, F20.89, F20.9 |
| Alcohol use disorder | ICD-9: 303, 303.01, 303.02, 303.03, 303.9, 303.91, 303.92, 303.93,  ICD-10: F10.20, F10.21, F10.220, F10.221, F10.229, F10.230, F10.231, F10.232, F10.239, F10.24, F10.250, F10.251, F10.259, F10.26, F10.27, F10.280, F10.281, F10.282, F10.288, F10.29 |
| Non-alcohol substance use disorder | ICD-9: 304, 304.0, 304.01, 304.02, 304.03, 304.08, 304.2, 304.21, 304.22, 304.23, 304.3, 304.31, 304.32, 304.33, 304.39, 304.4, 304.40, 304.41, 304.42, 304.42, 304.43, 304.43  ICD-10: F15.20, F15.21, F15.220, F15.221, F15.222, F15.229, F15.23, F15.24, F15.250, F15.251, F15.259, F15.280, F15.281, F15.282, F15.288, F15.29, F15.90, F15.920, F15.921, F15.922, F15.929, F15.93, F15.94, F15.950, F15.951, F15.959, F15.980, F15.981, F15.982, F15.988, F15.99, F12.20, F12.21, F12.220, F12.221, F12.222, F12.229, F12.250, F12.251, F12.259, F12.280, F12.288, F12.29, F14.20, F14.21, F14.220, F14.221, F14.222, F14.229, F14.23, F14.24, F14.250, F14.251, F14.259, F14.280, F14.281, F14.282, F14.288, F14.29, F11.20, F11.21, F11.220, F11.221, F11.222, F11.229, F11.23, F11.24, F11.250, F11.251, F11.259, F11.281, F11.282, F11.288, F11.29 |

*Note*. CPT=Current Procedural Technology; CVD=cardiovascular disease; ICD=International Classification of Diseases.

^a^Female-specific risk factors included gestational hypertension and diabetes, placental disorders, and preeclampsia

^b^Neuroendocrine disorders included hypothyroidism, hyperthyroidism, and adrenal and pituitary disorders
